# Outcomes of Kaposi sarcoma in people living with HIV who maintain long‐term viral suppression: A prospective case‐series

**DOI:** 10.1111/hiv.70280

**Published:** 2026-07-08

**Authors:** S. Bacquart, R. Palich, M. Veyri, S. Bregigeon‐Ronot, M.‐C. Picot, C. Vidal, L. Galicier, S. Choquet, J.‐P. Spano, M.‐A. Valantin, A. Makinson

**Affiliations:** ^1^ CHU Montpellier, INSERM U 1175, 191 avenue du Doyen Gaston Giraud Montpellier France; ^2^ Pitié Salpetrière AP‐HP, NSERM U1136, 27 rue Chaligny Paris France; ^3^ Pitié Salpêtrière, AP‐HP Institut Universitaire de Cancérologie, INSERM U1136 Paris France; ^4^ Service d'Immuno‐hématologie Clinique AP‐HM, INSERM, Hôpital Sainte‐Marguerite Université Aix Marseille, 270 boulevard de St Marguerite Marseille France; ^5^ CHU de Montpellier Unité de Recherche Clinique and Épidémiologie Montpellier France; ^6^ Hôpital La Timone, Médecine Interne, APHM, Université Aix Marseille Marseille France; ^7^ Hématologie, APHP Pitié‐Salpêtrière, 47 boulevard de l'hôpital Paris France

**Keywords:** AIDS, antiretroviral, cancer, HIV, Kaposi's sarcoma

## Abstract

**Background:**

Data on chemotherapeutic efficacy and tolerability for Kaposi Sarcomas (KS) in people living with HIV (PWH) and HIV‐virological control are limited. We aimed to describe clinical responses and tolerability of KS therapies in PWH with HIV‐aviraemic KS in a prospective cohort.

**Methods:**

We analysed all PWH with active KS with an HIV plasma viral load <200 copies/mL for more than 12 months under combination antiretroviral therapy (cART), whose clinical conditions were presented between the 1st of June 2021 and the 2nd of January 2023 to a national French Cancer HIV network panel. Clinicians completed a standardized questionnaire to assess KS history and standardized treatment response.

**Results:**

We included 18 PLHIV (94% male, median age 49 years). The median CD4 count was 614/mm3 and the median CD4/CD8 ratio was 0.8. Fifteen had already experienced relapses of KS. Median follow‐up time was 30.1 months (IQR [24 to 34.2]). We observed seven well‐controlled PWH with no KS relapse after a median follow‐up of 16 months (IQR 9.5 to 22.5), eight well‐controlled PWH who experienced KS relapse after a median of 6 months (IQR, 3–12) following chemotherapy, primarily limited to the skin, and two PWH with visceral KS relapse in a context of non‐adherence. PWH who relapsed/progressed were older and had a longer duration of HIV virological control.

**Conclusions:**

KS in PWH with virological control respond to chemotherapy, but are prone to relapses, primarily to the skin, and strategies can be limited by past and cumulative toxicities. Older age and longer duration of virological control may negatively impact the probability of KS response.

## INTRODUCTION

Kaposi's sarcoma (KS) is a low‐grade vascular tumour related to Kaposi sarcoma *herpesvirus/human herpesvirus* 8 (KSHV/HHV8) infection. In people living with HIV (PWH), the main entity is an AIDS classifying disease epidemic AIDS‐KS triggered by HIV replication and immunodeficiency [1], with reduced incidence since widespread use of combination antiretroviral therapy (cART) [2].

There have been recent descriptions of KS occurring in HIV well‐controlled PWH in retrospective series [3, 4, 5, 6]. In a study recollecting all clinical presentations of AIDS KS in PWH and endemic KS (HIV negative) from one French region, PWH with negative HIV viral load and KS disease presentations were generally more indolent than epidemic AIDS‐KS [5], except for younger age at occurrence. However, aggressive KS in well‐controlled PWH does occur [5, 6], encountering additional treatment challenges, as KS therapeutic options are limited and exclude the short‐term benefits of immune restoration and HIV‐virological control with cART. All these studies were however retrospective, impeding proper assessment of clinical responses and time to relapses.

We thus aimed in this study to prospectively describe clinical response and relapse of KS in PWH with controlled HIV‐viremia undergoing chemotherapy and referred to the French national Cancer VIH expert panel.

## METHODS

### Study design

This study is based on prospective data obtained from the French CancerVIH network, constituted of French experts in infectious diseases, oncology, radiotherapy and pharmacology, who virtually meet twice a month in a multidisciplinary team meeting (MDT) to optimise management of PWH with hard‐to‐treat cancers.

### Population

All PWH with HIV virological control (plasma viral load <200 copies/mL for more than 12 months) and KS, presented to the Cancer HIV MDT between the 1st of June 2021 and 2nd of January 2023, were included. Blips, defined by a transient increase in HIV‐viral replication <1000 copies/mL, were allowed.

### Baseline data collection and follow‐up

PWH were followed up until death, date of loss to follow‐up, or 30th of June 2024, whatever occurred first. Clinicians completed a standardized questionnaire during an interview with the main author (SB) to recollect demographic and immunovirological data, prior KS episodes, number of cures, treatment response and tolerance. Baseline characteristics were reported using median and interquartile range (IQR) for continuous variables and frequencies and proportions for categorical variables.

The referent physician was thereafter contacted every 6 months to assess for each case treatment response and tolerance.

### Outcomes

The main author and the PWH's referral doctor classified responses using the ACTG response outcomes for epidemic AIDS‐KS [7]. KS progression was defined as the appearance of new KS lesions under treatment; KS relapse was defined as the appearance of new lesions leading to the reinitiation of treatment after initial response and KS treatment termination; partial response as the persistence of sequela KS lesions with no new lesions; and complete response as the disappearance of KS lesions, clinically or radiologically assessed when necessary. No statistical tests were performed due to the small sample size.

The CancerVIH database was approved by the French Institutional Evaluation Committee (number 15‐009), the French Advisory committee on the processing of health data (number 16‐391) and the French Data Protection Authority (CNIL) (number 916500).

## RESULTS

A total of 18 PWH with KS and well‐controlled HIV were presented to the CANCERVIH network. Description of these PWH is presented in Table 1. Briefly, PWH were predominantly male (94%), with a median age of 49 years, IQR [41 to 56] and a median HIV diagnosis history of 15 years, IQR [4 to 25]. Fifteen had a history of AIDS‐KS with HIV‐replication, 15 a history of at least one prior episode of KS despite HIV control (median of two relapses) and 3 were de novo KS cases. The median CD4 nadir was 282 cells/mm^3^ (IQR [156 to 312]), the median CD4 count at inclusion was 614 cells/mm3 (IQR [479 to 871]) and the median CD4/CD8 ratio was 0.8 (IQR [0.60 to 1.13]). HIV‐virological control had been achieved for a median of 11 years (IQR [2 to 14]). Median time between the end of last KS chemotherapy and presentation at MDT was 12 months (IQR [4 to 20]). Prior KS therapies are detailed in Table 1.

**TABLE 1:** Patient characteristics at inclusion ^1^T1: Tumour‐associated edema or ulceration, extensive oral KS, KS in viscera; I1: CD4+ cell count <200/mm^3^; S1: History of opportunistic infection, ‘B’ symptoms present, Karnosky performance status <70. KS: Kaposi sarcoma. TIS score: Tomical features of the lesions (T), immunological assessment (I) and HIV‐related systemic illness (S).

	*N* (%) or median, IQR
Age (years)	49 [41, 56]
Male at birth	17 (94%)
Time since HIV diagnosis (in years)	15 [4, 25]
Time since virological suppression (in years)	11 [2, 14]
Nadir CD4	282 [156, 312]
History of viremic epidemic AIDS KS	15 (83%)
CD4 count at inclusion	614 [479, 871]
Ratio CD4/CD8	0,8 [0.60, 1.13]
Time since KS diagnosis (in years)	4 [1; 21]
History of relapses since HIV aviremia	2 [1, 3]
0 (no relapse, first episode)	3 (16.7%)
1	8 (50%)
2	2 (11%)
≥3	5 (28%)
Strict skin involvement	14 (78%)
Other KS Visceral locations	4 (22%)
Digestive	4 (22%)
Bone	1 (5.6%)
Mucous	1 (5.6%)
Pulmonary	4 (22%)
Ganglion	4 (22%)
Kriegel score	
I (locally indolent KS cutaneous lesions)	10 (55%)
II (locally invasive and aggressive form)	4 (22%)
III (disseminated mucocutaneous form, often with lymph node involvement)	0
IV (disseminated, mucocutaneous form with visceral involvement)	4 (22%)
TIS score^a^	
T1	5 (28%)
I1	0
S1	5 (28%)
History of treatment with antracyclines	13 (72%)
If yes, number of total administrations	6 [6, 15]
Response at last cure (partial or complete)	12 (92%)
Failure	1 (8%)
Treatment interruption for intolerance (cardiac *n* = 1), vocal cord paresis (*n* = 1), …(*n* = 1)	3 (23%)
History of treatment with taxanes	8 (44%)
If yes, number of administrations	13 [8.25, 22]
Response at last cure (partial or complete)	8 (100%)
Failure	0
Treatment interruption for intolerance	0 (but 2 intolerance)
Other prior treatments (chemotherapy or local)	9 (50%)
Other chemotherapy^a^	7 (38.9%)
Local treatment	4 (22%)

^a^ Bleomycin (*n* = 4), vinblastine (*n* = 1), interferon (*n* = 1), imatinib (*n* = 1).

At study entry, 14 (78%) PWH had skin lesions only, including all 3 KS de novo cases, 7 (39%) presenting with more than 20 skin lesions (including 1 de novo case). One PWH was lost to follow‐up due to relocation to another country. Median follow‐up time of the remaining 17 PWH was 30.1 months (IQR [24 to 34.2]) after MDT presentation. Clinical evolution and treatment response of each PWH during follow‐up is shown in Table 2.

**TABLE 2:** Clinical follow up and treatments received and response during follow‐up.

Characteristic	*N* = 17^a^
Duration of follow‐up (months)	30.1 (24.0, 34.2)
Death during follow up	3 (18%)
From KS	2 (12%)
From decompensated liver disease	1 (6%)
PWH with relapses or KS treatment failures during follow‐up	10 (59%)
With maintenance of HIV aviremia	8 (47%)
Chemotherapy failure with HIV viremia	2 (12%)
Time between last course of chemotherapy and first relapse (months)	
Median, IQR	6 [3, 12]
<6 months between end of chemotherapy and progression	4
Between 6 and 12 months	2
>12 months	0
Number of relapses or failures with undetectable viral load, leading to renewed treatment (*n* = 8)	
1	3 (18%)
2	3 (18%)
3	1 (5.9%)
4	1 (5.9%)
New or evolving visceral lesion	0 (0%)
Chemotherapy treatment	14 (82%)
Anthracyclines	7 (41%)
Number of administrations (IQR)	6.0 [6.0, 11.5]
Response at last cure	
Failure	1 (14%)
Partial or complete response	5 (71.4%)
Discontinuation by the patient	1 (14%)
Intolerance	3 (42.8%): neutropenia, anorexia, ageusia
Relapses	4 (24%)
Median time to relapse (months)	4.5 [1, 6]
Taxanes	10 (59%)
Number of total administrations	11 [6, 23]
Response at last cure	
Failure	1 (10%)
Partial or complete response	9 (90%)
Intolerance	3 (33.3%): neuropathy
Relapses	6 (33%)
Median time to relapse (months)	6 [4, 8]
Other treatments	
Cryotherapy	1 (5.9%)
Topical fluorouracil	1 (5.9%)
Radiotherapy	2 (11.8%)
Pomalidomide	2 (11.8%)
Pembrolizumab	1 (5.9%)

^a^ *n* (%); median [IQR].

We described three clinical patterns during follow‐up, according to KS response to treatment and HIV‐viral load:

1. 7 PWH with KS partial response after a median follow‐up of 16 months (IQR [9.5 to 22.5]) after end of chemotherapy did not relapse. All maintained HIV viral load control, though 2 had transient viremia <6 months (100 and 19 000 copies/mL). Three were treated with anthracyclines (for 4, 6 and 14 months, with 4, 6 and 21 administrations, respectively) and two with taxanes (for 12 and 6 months, with 23 and 11 administrations, respectively). One PWH followed his taxane therapy with pomalidomide 5 mg/day for 21 days each month for 12 months. One PWH received topical fluorouracil only.
2. Eight PWH relapsed or progressed during their follow‐up despite HIV controlled viral load after a median follow‐up time of 31 months, IQR [24 to 34]. Only one had visceral KS lesions, and 7 locally indolent or locally invasive KS. Five of the eight PWH had more than one relapse: three had two relapses, one had three relapses and one had four relapses. All relapses were limited to the skin. Three were treated with anthracyclines (median of 6 administrations [6 to 9]) and seven with taxanes (median of 6 administrations [6 to 25]), and three PWH received both. One was treated with local radiotherapy only. One PWH died after multiple relapses after 30 months of follow‐up, but the cause of death was linked to advanced metabolic cirrhosis, without evidence of KS related liver damage. He was treated by 20 courses of anthracyclines, 25 courses of taxanes, 12 months of pomalidomide followed by 6 courses of pembrolizumab. Nevertheless, KS progressed under pembrolizumab, justifying the reintroduction of paclitaxel, which led to improvement in his lesions.
3. Two PWH relapsed following loss of virological control due to cART interruption. These PWH were lost to follow‐up for 6 and 14 months, with HIV viral loads of 240 000 copies and 316 000 copies/mL at readmission, respectively. Both PWH died with active visceral KS, one with diffuse KS skin lesions and probable digestive and pulmonary involvement and the other with significant lung lesions. Both died as a result of respiratory distress, and one suffered neurological deterioration.

In PWH who maintained a controlled HIV viral load during follow‐up, PWH who relapsed had longer duration of HIV virological control versus PWH who did not relapse (median of 12 years (IQR [6, 8]) versus 3 years (IQR [1.5 to 7.5])). Moreover, PWH who relapsed were older than responders (median age 54 years (IQR [43 to 54]) versus 44 years (IQR [37 to 55])). Numbers of previous relapses prior to the prospective follow‐up were similar in both groups.

We compared the CD4 count and the CD4/CD8 ratio in PWH who received standard treatments during the prospective follow‐up. There was no significant difference between the start and end of the follow‐up period. At the end of the follow‐up period, the median CD4 count among PWH treated with chemotherapy was 700/mm^3^, IQR [475 to 921] and the CD4/CD8 ratio was 0.8 [0.64 to 1.05], compared to 649/mm3, IQR [478–840] for CD4, and 0.8 [0.60 to 1.27] at baseline.

During follow‐up, anthracyclines treatment was poorly tolerated in 3 PWH (43%), leading to discontinuation due to neutropenia, anorexia, or ageusia. Taxane was discontinued in 3 PWH due to neuropathy.

## DISCUSSION

In this study, we described 18 PWH with KS and well controlled HIV‐viremia referred to the French national MDT CancerVIH expert panel for therapeutic advice. We found that most cases had a history of epidemic AIDS and relapses despite controlled HIV viral load, though de novo cases in well‐controlled PWH occurred in a minority of instances. We also objectified that most cases at presentation and all cases at relapses were limited to the skin, although diffuse skin lesions (more than 20) were not uncommon and one had visceral involvement, and that 7 PWH with KS partial response did not relapse during the prospective follow‐up.

The proportion of PWH with visceral lesions at inclusion is in line with the observational study conducted by the CancerVIH group [6], but higher than other retrospective cohorts [3, 4]. The number of PWH who experienced relapses varies across retrospective studies: 35% in the study by Mani et al. [4], 62% in the CancerVIH group cohort [6], which is consistent with our study (58% of PWH). Our case‐series study is not representative of HIV well‐controlled PWH and KS, due to a referral bias to the MDR, where only the most severe cases are presented.

Our study also shows that treatment of KS in well‐controlled PWH is effective but prone to relapses and cumulative toxicities. Our patterns of KS therapy response show that maintaining HIV‐virological control is paramount to maintaining response throughout follow‐up (pattern 1) or to limit recurrences to primarily skin lesions.

There are no data in the literature regarding the toxicity of repeated courses of chemotherapy in this specific population. Regarding anthracyclines, the main risk is heart failure; dysaesthesia and bone marrow toxicity may also occur [9]. Taxanes have been designated as second‐line treatments due to their poorer tolerability, particularly in terms of the risk of peripheral neuropathy, cytopenia and alopecia [10, 21]. In our cohort, poor tolerance was reported in 42% of PWH receiving anthracyclines and 33% of those receiving taxanes, which highlights the need to develop new treatment regimens.

The pathophysiological mechanisms underlying KS relapses despite HIV virological control remain hypothetical. Chronic antigenic stimulation by HHV‐8 and persistence of HIV viral activity within the tumour [11] contribute to chronic immune dysfunction, in PWH with probable incomplete qualitative immune restoration [12]. Indeed, persistent activation of dysfunctional CD8+ T lymphocytes has been observed [13]. Furthermore, a study has shown that an immunosenescence phenotype (expansion of CD57+ and CD28‐ T lymphocytes) is associated with the development of KS in PWH receiving effective antiretroviral therapy [14]. The role of NK lymphocytes has also been hypothesized, with evidence of hyporeactivity [15]. Thus, KS in well‐controlled PWH appears to result from the interplay of two persistent factors: low‐grade viral HHV‐8 infection and a pre‐existing, self‐maintaining immune dysfunction, potentially aggravated by immunosenescence. Interestingly, under chemotherapy, CD4 and CD4/CD8 ratio remained stable, implying that chemotherapy was probably not responsible for the perpetuation of KS in these PWH.

We found that PWH with relapses/progression had a numerically longer duration of HIV virological control (12 years) versus PWH who did not (3 years), despite having similar levels of CD4 levels, CD4/CD8 ratio, nadir CD4 and histories of AIDS events. We also observed older ages in PWH who relapsed during follow‐up, potentially reflecting the impact of immune senescence on HHV‐8 proliferation. These findings need to be evaluated in larger cohort studies but could reflect enhanced functional immune impairment in this population.

Whether some PWH could further benefit from KS ‘maintenance’ therapies – such as long term pomalidomide, interferon or immunomodulatory treatments – remains to be assessed in prospective studies. There is indeed a need for less toxic treatments of KS to mitigate relapses. Among possibilities, pomalidomide, through its anti‐angiogenic properties and T lymphocytic stimulation, was evaluated in a Phase I/II trial in PWH or not, and without visceral involvement, achieving a partial and complete response rate of 71% (95% CI, 51%–87%) [16]. Pomalidomide, as a rescue therapy, was ineffective in one PWH in our case‐series and appeared effective in another. Anti‐PD1 and PDl‐1 immunotherapy is currently being explored, especially as spindle cells express PD‐1 to escape the immune system [17]. Published case series [8, 18, 19] have shown some efficacy. A recent study of 30 PWH with KS despite controlled HIV viremia showed a response rate of 62.1%, with 31% experiencing immune‐mediated reaction and 25% requiring the addition of corticosteroid therapy [20]. Only one PWH in our cohort received immunotherapy, without evident efficacy.

Our study has limitations. Baseline data were collected through interviews with referring doctors, which may introduce reporting bias. However, these data were retrieved from electronic clinical records, limiting memory biases. Numbers of cases were limited, and the profiles and management of PWH were heterogeneous, which impeded the reliability of statistical comparisons. As such, our conclusions must be interpreted with caution. Also, follow‐up did not standardise protocols and lengths of chemotherapy administration. However, as a prospective study, we provided valuable data during the follow‐up period, particularly regarding therapeutic responses and relapses.

In conclusion, our case‐series show that cases of KS in PWH with HIV‐controlled have complex management strategies and first line chemotherapies appear to be effective in the short‐term, though patients are prone to relapse, commonly limited to the skin. Longer duration of HIV virological control prior and older age are factors potentially associated with KS relapse, as well as the effectiveness of KS ‘maintenance’ therapies in PWH with frequent relapses need to be investigated in larger cohorts.

## AUTHOR CONTRIBUTIONS

The authors thank all investigators and staff of the French CancerVIH Network for their contribution to PWH care and data collection. S. Bacquart collected the data, performed the statistical analyses, and wrote the first draft of the manuscript. A. Makinson, R Palich, M‐A Valantin contributed to data interpretation and manuscript writing. M‐C Picot and C Vidal performed the statistical analyses. The remaining authors reviewed the manuscript and provided critical feedback.

## Data Availability

The data that support the findings of this study are available from the corresponding author upon reasonable request.
